# Metabolic Dysfunction-Associated Steatohepatitis With Positive Antimitochondrial M2 Antibodies: Diagnostic Pitfall and Importance of Follow-Up

**DOI:** 10.7759/cureus.99347

**Published:** 2025-12-16

**Authors:** Sara Mounsif, Omar Bahlaoui, Hanane Delsa, Wafaa Khannoussi, Imane Ben El Barhdadi

**Affiliations:** 1 Gastroenterology and Hepatology, Cheikh Khalifa International University Hospital, Mohammed VI University of Sciences and Health, Casablanca, MAR; 2 Research Unit, Mohammed VI Center for Research and Innovation, Rabat, MAR

**Keywords:** alkaline phosphatase (alp), anti-mitochondrial antibody, metabolic dysfunction-associated steatohepatitis (mash), non-alcoholic steatohepatitis, primary biliary cholangitis (pbc)

## Abstract

Metabolic dysfunction-associated steatohepatitis (MASH) is a subtype of metabolic dysfunction-associated steatotic liver disease (MASLD) characterized by liver fat accumulation and inflammation. Occasionally, patients with MASH may test positive for antimitochondrial M2 antibodies (AMA-M2), which are usually linked to primary biliary cholangitis (PBC), creating potential diagnostic confusion.

We report the case of a 52-year-old woman with obesity and persistent liver enzyme elevation, who was found to have positive AMA-M2 antibodies. Imaging revealed moderate hepatic steatosis, and liver biopsy confirmed MASH without evidence of PBC. After adopting lifestyle modifications, the patient achieved significant weight loss and normalization of liver and lipid profiles. At the one-year follow-up, the patient showed isolated elevation of alkaline phosphatase, which, together with positive anti-M2 antibodies, led to a diagnosis of PBC and initiation of ursodeoxycholic acid therapy. This case illustrates that anti-M2 antibodies may be found in patients with MASH alone or in those with MASH associated with PBC, highlighting the importance of careful evaluation and long-term follow-up.

## Introduction

Metabolic dysfunction-associated steatotic liver disease (MASLD), formerly known as non-alcoholic fatty liver disease (NAFLD), is a spectrum of liver conditions characterized by hepatic steatosis and at least one cardiometabolic risk factor (among overweight, obesity, type 2 diabetes, hypertension, hypertriglyceridemia, or low high-density lipoprotein (HDL) cholesterol) in the absence of significant alcohol consumption (<20 g/day for women, <30 g/day for men) [1]. It has become the leading cause of chronic liver disease worldwide, affecting more than one-third of the adult population [2].

Metabolic dysfunction-associated steatohepatitis (MASH), formerly known as non-alcoholic steatohepatitis (NASH), is a progressive subtype of MASLD defined by steatosis with liver inflammation and hepatocellular injury. It is often asymptomatic, and its definitive diagnosis relies on liver biopsy. However, MASH can occasionally present with positive antimitochondrial M2 antibodies (AMA-M2), which may complicate the differential diagnosis with primary biliary cholangitis (PBC). We present a case of MASH associated with positive AMA-M2.

## Case presentation

A 52-year-old female patient with no medical or surgical history was referred to our hepatology consultation by her primary care physician for the evaluation of chronic liver enzyme elevation. On questioning, the patient reported chronic fatigue. She had no symptoms of cholestasis, such as pruritus or jaundice, and no signs of alopecia, rash, or joint pain. She denied alcohol consumption, drug use, medications, or herbal remedies. There were no known connective tissue disorders or thyroid diseases. She had no personal history of diabetes and no family history of liver disease, autoimmune diseases, thyroid disorders, or diabetes.

Physical examination revealed moderate obesity with a body mass index of 36.5 kg/m² and a weight of 89 kg. The rest of the examination was otherwise normal, showing no stigmata of chronic liver disease or abdominal mass.

Serial liver function tests showed persistent elevation of liver enzymes. Alanine aminotransferase (ALT) was twice the upper limit of normal, and aspartate aminotransferase (AST) was 1.6 times the upper limit of normal. There was no cholestasis, with normal alkaline phosphatase (ALP) and gamma-glutamyl transferase, and no evidence of hepatic synthetic dysfunction, with prothrombin time and albumin levels within normal limits. The lipid profile revealed elevated levels of total cholesterol, low-density lipoprotein (LDL), and triglycerides. The glycemic workup, including fasting blood glucose and glycated hemoglobin, was within normal limits. Total immunoglobulin levels were also normal. The results of these assessments are summarized in Table 1.

**Table 1:** Laboratory workup results

	Value	Reference range
Alanine aminotransferase (ALT)	107 U/L	<55
Aspartate aminotransferase (AST)	55 U/L	5-34
Gamma-glutamyl transferase (GGT)	50 U/L	<55
Alkaline phosphatase	99 U/L	40-150
Total bilirubin	7 mg/L	2-12
Prothrombin time (PT)	12 seconds	10-13
Prothrombin time ratio	99.6%	70-100
Albumin	41 g/L	34-50
Total cholesterol	2.93 g/L	<2
Low-density lipoprotein (LDL)	2.10 g/L	<1.9
Triglycerides	1.97 g/L	<1.5
Fasting blood glucose	0.99 g/L	0.7-1.1
Glycated hemoglobin (HbA1c)	5%	<5.7
Total immunoglobulin G (IgG)	12 g/L	7-16

**Figure 1:**
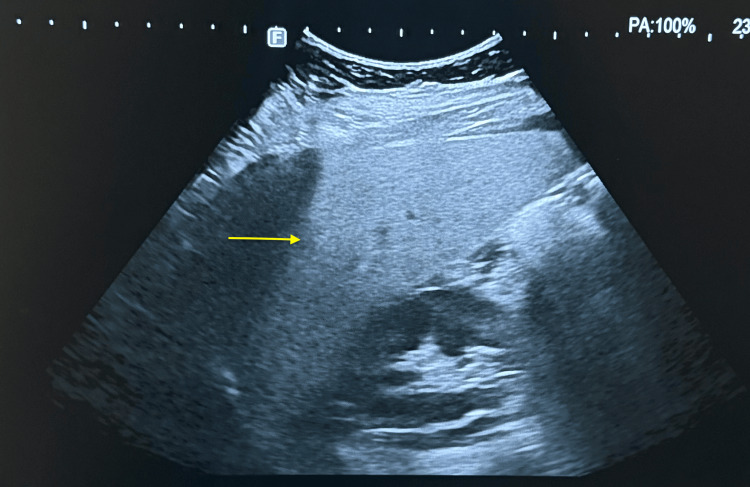
Ultrasound findings of hepatic steatosis Ultrasound image showing increased hepatic echogenicity compared with the right kidney, consistent with steatosis (yellow arrow indicating the liver).

An abdominal ultrasound with Doppler imaging was performed and revealed stage 2 hepatic steatosis (Figure 1).

The diagnosis of MASLD was established. Viral hepatitis was ruled out, with negative serologies for hepatitis A, B, and C. Immunofluorescence testing revealed positive antinuclear antibodies (ANA) at a titer of 1:640, with a cytoplasmic pattern suggestive of antimitochondrial antibodies (AMA). Western blot detected strongly positive AMA (M2 and M2-3E) with intensities of 136 and 157, respectively. Antibodies against sp100, gp210, smooth muscle, and PML tested negative.

An ultrasound-guided liver biopsy was performed and revealed chronic cytolytic and fibrosing hepatitis with minimal micro- and macrovesicular steatosis (15%) and ballooning of hepatocytes, suggestive of MASH. The SAF (steatosis, activity, and fibrosis) score was S1A2F2. There were no histological signs of autoimmune hepatitis, cholangitis, or siderosis.

**Figure 2:**
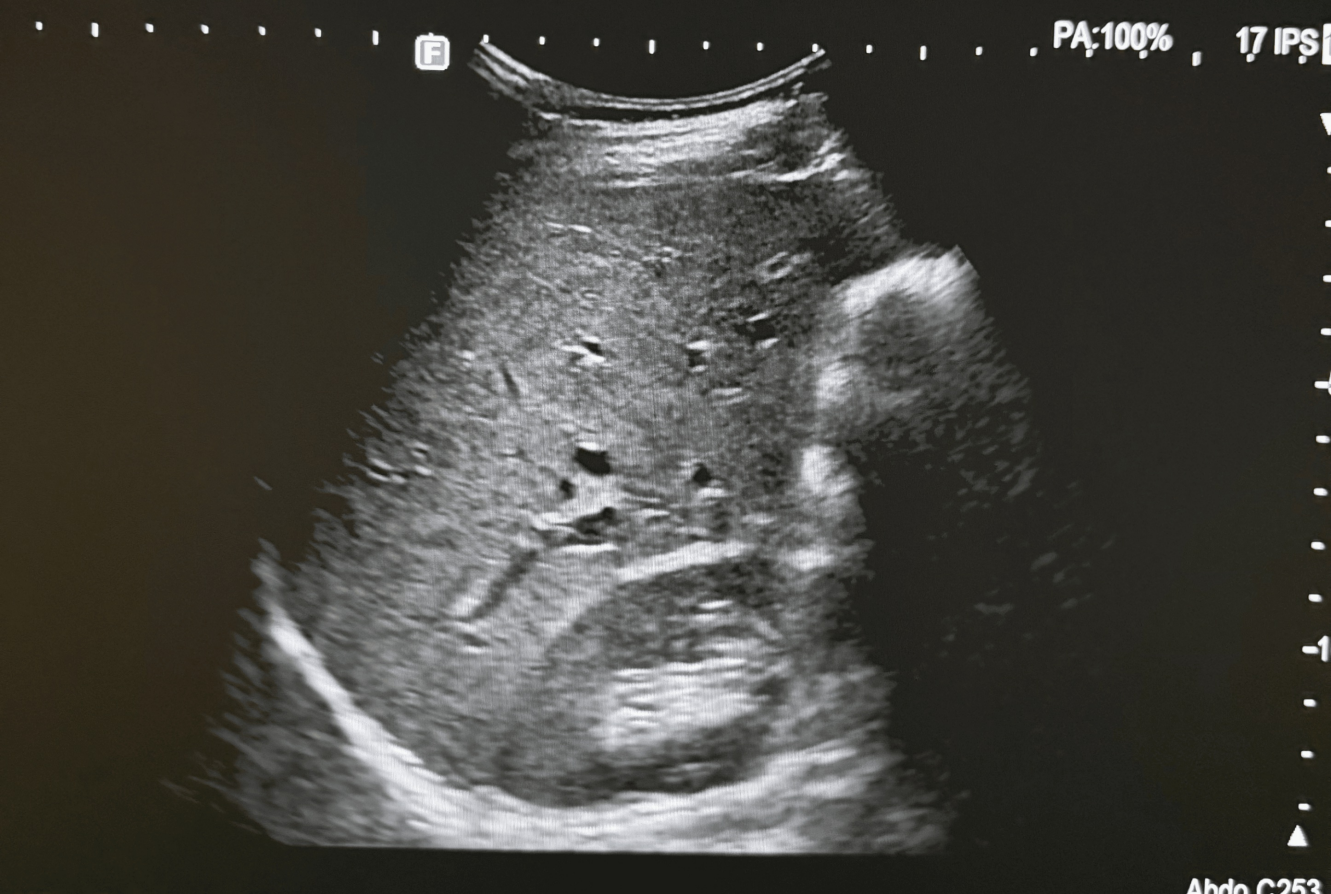
Abdominal ultrasound at 6-month follow-up The ultrasound shows normal liver and kidney appearance.

The patient was advised to follow a dietary regimen and engage in regular physical activity, with a goal of losing 10% of her body weight. She was monitored clinically and with laboratory tests. At the six-month follow-up, the patient weighed 61 kg with a body mass index of 25.4 kg/m². Her hepatic and lipid profiles were within normal limits, as shown in Table 2, and her abdominal ultrasound was unremarkable (Figure 2).

**Table 2:** Six-month follow-up laboratory assessment

	Value	Reference range
Alanine aminotransferase (ALT)	17 U/L	<35
Aspartate aminotransferase (AST)	15 U/L	<35
Gamma-glutamyl transferase (GGT)	20 U/L	<32
Alkaline phosphatase	96 U/L	42-98
Total cholesterol	1.92 g/L	<2
Low-density lipoprotein (LDL)	1.12 g/L	<1.9
Triglycerides	0.88 g/L	<1.5

**Figure 3:**
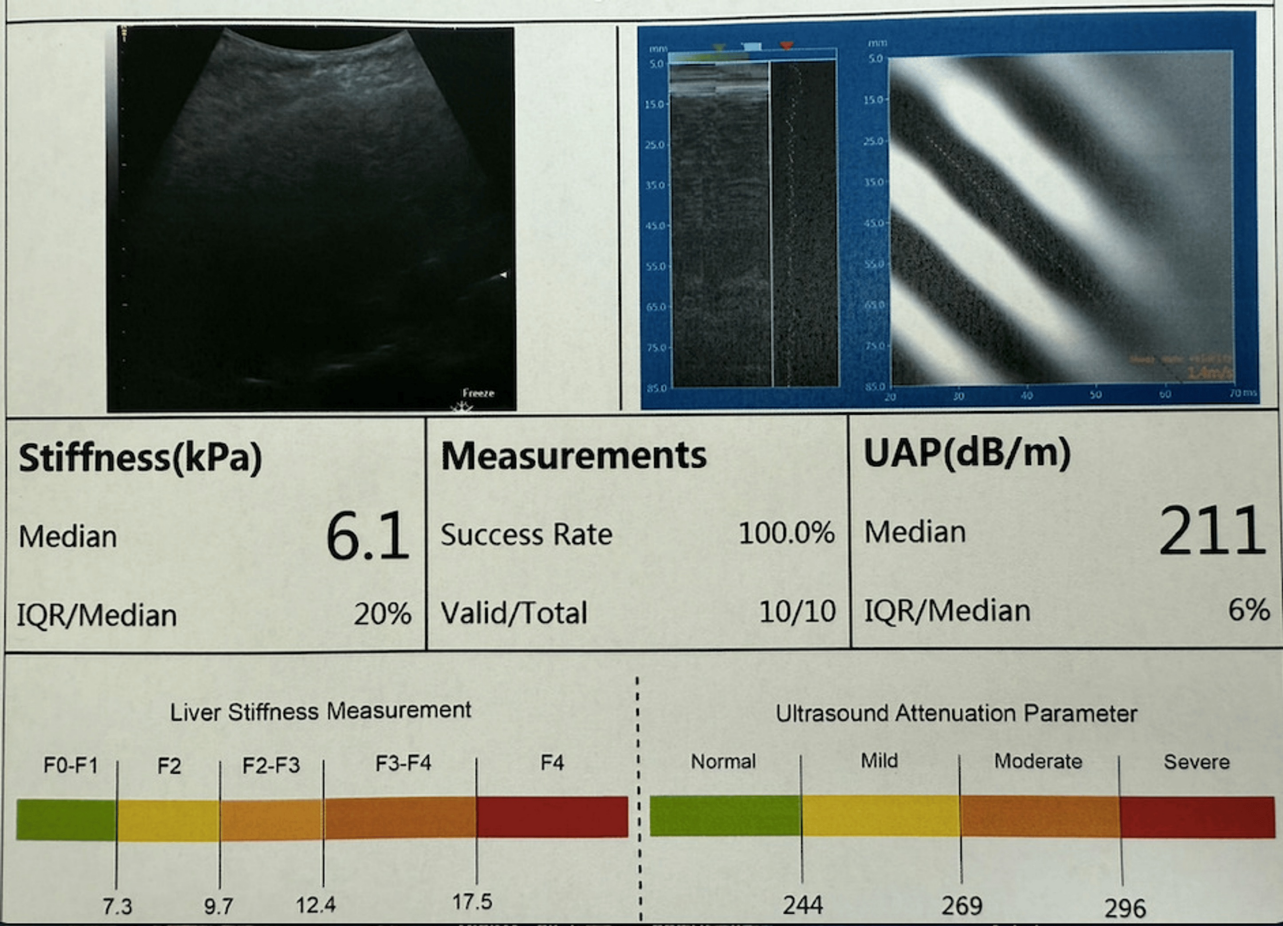
Liver elastography at one year Liver elastography showed F0 fibrosis with a stiffness of 6.1 kPa and a normal ultrasound attenuation parameter of 211 dB/m, indicating the absence of hepatic steatosis.

At the one-year follow-up, liver elastography indicated F0 fibrosis with a stiffness measurement of 6.1 kPa (Figure 3). 

Liver function tests were monitored and showed elevated ALP levels at 225 IU/L, while the remaining tests, including gamma-glutamyl transferase, total bilirubin, and transaminases, were within normal limits, as shown in Table 3.

**Table 3:** Liver function tests at 1-year follow-up

	Value	Reference range
Alanine aminotransferase (ALT)	12 U/L	<35
Aspartate aminotransferase (AST)	14 U/L	<35
Gamma-glutamyl transferase (GGT)	18 U/L	<32
Alkaline phosphatase	225 U/L	42-98
Total bilirubin	5 mg/L	<12

The diagnosis of PBC was established based on positive anti-M2 antibodies and elevated ALP levels. The patient was started on ursodeoxycholic acid at a dosage of 14.7 mg/kg/day. At the four-month follow-up, ALP levels had improved to 1.3 times the upper limit of normal (125 U/L) compared to 2.3 times at diagnosis (225 U/L), and no cytolysis was observed.

## Discussion

MASH is a chronic disease resulting from the interaction of metabolic and environmental factors (such as type 2 diabetes, obesity, physical inactivity, and menopause). It is marked by two key histological features: hepatocyte ballooning and lobular inflammation. While liver biopsy is not routinely recommended, it remains the definitive method for confirming a MASH diagnosis [1].

Clinically, MASH is often asymptomatic and is suspected when elevated liver enzymes are observed alongside radiological signs of steatosis. It can lead to hepatic complications, including cirrhosis and hepatocellular carcinoma, and extrahepatic complications, including cardiovascular events and other cancers.

Therapeutic options for MASH remain limited. Weight loss is the primary treatment and is recommended for all cases of MASH, including those with normal body weight. It can result in a reduction in steatosis (≥5% weight loss), a decrease in hepatic inflammation (7%-10%), and a reduction in fibrosis (≥10%) [1]. To achieve this weight loss goal, several strategies can be considered: lifestyle and dietary changes, medications such as GLP-1 receptor agonists, or bariatric surgery in specific indications [1]. Resmetirom is currently the only recommended treatment specifically targeting MASH. It may be considered in cases of non-cirrhotic MASH with significant fibrosis (≥F2) [1]. However, it is not available in most countries, and studies on its long-term efficacy are still ongoing.

Given its high prevalence, MASH may coexist with other chronic liver diseases. Therefore, it is necessary to rule out other causes of liver disease, particularly using blood tests, including autoimmune screening. Rare cases of MASH with positive AMA-M2 without PBC have been reported [3]. This poses a diagnostic challenge with PBC, particularly as cases of PBC associated with MASH have also been reported [4,5]. These cases of overlap between MASH and PBC require tailored management due to the increased risk of progression to cirrhosis [6].

AMA are autoantibodies directed against specific mitochondrial proteins. They include subtypes such as anti-M2, anti-M1, anti-M3, and anti-M4, each targeting different mitochondrial antigens. AMA-M2 are particularly associated with PBC. They are primarily detected by indirect immunofluorescence (minimum diagnostic titer 1:40) or Western blot using M2 or M2-3E antigens. They are a key test for diagnosing PBC, with a sensitivity and specificity of 84% and 98%, respectively [7]. The presence of intrahepatic cholestasis along with positivity for these autoantibodies is sufficient to diagnose PBC, so liver histology is not required [8].

The prevalence of positive AMA-M2 in the general population is relatively high (up to 1/1,000) [9], but in the absence of cholestasis, their diagnostic value is very low [10]. Multiple studies have shown that the detection of these antibodies can precede the onset of PBC by several years, and that one in six patients with positive AMA-M2 and normal ALP levels will develop PBC within five years [11]. Prolonged follow-up is therefore required, and it is recommended to monitor these patients with annual liver function tests [12].

Also, studies have reported the presence of autoimmune markers (ANA, AMA, and anti-smooth muscle antibodies (ASMA)) in MASLD at a higher prevalence than in the general population, ranging from 12% to 48% [3]. In a cohort of 398 NAFLD patients, 1% were AMA-M2-positive without PBC or other autoimmune diseases, showing that such antibodies can occasionally occur in MASLD alone [13]. This increased prevalence may be attributed to liver inflammation caused by MASH, which could result in bile duct damage, potentially leading to immunological cross-reactivity and stimulating AMA production. However, positivity for autoimmune markers (AMA, ANA, or ASMA) was not associated with differences in the clinical presentation or progression of MASLD [13].

## Conclusions

Positive AMA-M2 can be present in MASH without PBC. However, as MASH may coexist with other liver diseases, it is important to rule out PBC in this context. These patients also require prolonged follow-up, as they may develop PBC in the future, as illustrated by our case.
